# Menstrual health: a definition for policy, practice, and research

**DOI:** 10.1080/26410397.2021.1911618

**Published:** 2021-04-29

**Authors:** Julie Hennegan, Inga T. Winkler, Chris Bobel, Danielle Keiser, Janie Hampton, Gerda Larsson, Venkatraman Chandra-Mouli, Marina Plesons, Thérèse Mahon

**Affiliations:** a Research Fellow, Maternal, Child and Adolescent Health Program, Burnet Institute, Melbourne, Australia; Adjunct Research Associate, Department of Environmental Health and Engineering, Johns Hopkins Bloomberg School of Public Health, Baltimore, MD, USA; b Lecturer in Human Rights, Institute for the Study of Human Rights, Columbia University, New York, NY, USA; c Professor, Women’s, Gender, and Sexuality Studies, College of Liberal Arts, Department of Women’s, Gender, and Sexuality Studies, University of Massachusetts Boston, Boston, MA, USA; d Founder & Executive Director, Menstrual Health Hub / MH Hub, Berlin, Germany; e Co-Founder, Menstrual Cup Coalition, Nairobi, Kenya; f Co-Founder and Managing Director, The Case for Her, Stockholm, Sweden; g Scientist, UNDP–UNFPA–UNICEF–WHO–World Bank Special Programme of Research, Development and Research Training in Human Reproduction (HRP), Department of Sexual and Reproductive Health and Research, World Health Organization, Geneva, Switzerland; h Consultant, UNDP–UNFPA–UNICEF–WHO–World Bank Special Programme of Research, Development and Research Training in Human Reproduction (HRP), Department of Sexual and Reproductive Health and Research, World Health Organization, Geneva, Switzerland; i Regional Programme Manager South Asia, WaterAid, London, UK.

**Keywords:** gender equality, health, menstrual cycle, menstrual health, human rights

## Abstract

The term “menstrual health” has seen increased use across advocacy, programming, policy, and research, but has lacked a consistent, self-contained definition. As a rapidly growing field of research and practice a comprehensive definition is needed to (1) ensure menstrual health is prioritised as a unified objective in global health, development, national policy, and funding frameworks, (2) elucidate the breadth of menstrual health, even where different needs may be prioritised in different sectors, and (3) facilitate a shared vocabulary through which stakeholders can communicate across silos to share learning. To achieve these aims, we present a definition of menstrual health developed by the Terminology Action Group of the Global Menstrual Collective. We describe the definition development process, drawing on existing research and terminology, related definitions of health, and consultation with a broad set of stakeholders. Further, we provide elaboration, based on current evidence, to support interpretation of the definition.

## Résumé

Le terme de « santé menstruelle » est de plus en plus utilisé dans le plaidoyer, la programmation, les politiques et la recherche, mais il lui manque une définition cohérente et indépendante. Ce domaine de recherche et de pratique enregistrant une croissance rapide nécessite une définition complète pour 1) garantir la priorisation de la santé menstruelle comme objectif unifié dans les cadres de la santé mondiale, du développement, des politiques nationales et du financement, 2) préciser l'étendue de la santé menstruelle, même lorsque différents besoins peuvent faire l'objet de priorités dans différents secteurs, et 3) faciliter un vocabulaire commun avec lequel les parties prenantes peuvent communiquer au-delà des cloisonnements pour partager l'apprentissage. Pour parvenir à ces objectifs, nous présentons une définition de la santé menstruelle préparée par le groupe d'action sur la terminologie du collectif mondial sur les menstruations (Global Menstrual Collective). Nous décrivons le processus d'élaboration de la définition, fondé sur la terminologie et les recherches existantes, les définitions apparentées de la santé, et des consultations avec un large éventail de parties prenantes. De plus, nous fournissons des justifications, sur la base des données actuelles, à l'appui de l'interprétation de la définition.

## Resumen

El uso del término *“salud menstrual”* ha aumentado en las áreas de promoción y defensa, programas, políticas e investigación, pero se carece de una definición sistemática y autocontenida. En un campo de investigación y práctica que continúa creciendo rápidamente, se necesita una definición integral para (1) garantizar que la salud menstrual sea priorizada como objetivo unificado en los marcos de salud mundial, desarrollo, políticas nacionales y financiamiento, (2) dilucidar la amplitud de la salud menstrual, aun cuando diferentes necesidades sean priorizadas en diferentes sectores, y (3) facilitar un vocabulario común con el cual las partes interesadas puedan comunicarse entre silos para intercambiar conocimientos. Para lograr estos objetivos, presentamos una definición de salud menstrual formulada por el Grupo de Acción en Terminología de la Colectiva Menstrual Mundial. Describimos el proceso de formulación de la definición, basándonos en investigaciones y terminología existentes, en definiciones de salud relacionadas y en consulta con una gran variedad de partes interesadas. Además, proporcionamos elaboración, basada en la evidencia actual, para apoyar la interpretación de la definición.

## Introduction

Menstrual health is integral to improving global population health,^1^ achieving the Sustainable Development Goals, and realising gender equality and human rights.^2,3^ Although the past decade has seen growing awareness of menstrual-related challenges,^4,5^ increased multi-sectoral investment is needed to comprehensively address the needs of all people who menstruate.

Research and practice have developed a nuanced understanding of menstrual experiences, and their intersections with physical, mental, and social health.^6,7^ Varied terminologies have evolved, but increasingly actors are using *menstrual health* to evoke a holistic framework relevant to the varied objectives of policy and programming. Despite broad usage, *menstrual health* lacks a formal, self-contained definition. This has complicated advocacy efforts and has led to fragmented action and funding for menstrual health as organisations struggle to conceptualise the topic and situate it within their mandates. A unified definition of menstrual health is thus needed to advance advocacy, policy, practice, and research, highlight the relevance of menstrual health across sectors, and facilitate communication across stakeholder groups.

## Developing a definition of menstrual health

This definition of menstrual health was developed through a multi-stage process, led by the Terminology Action Group of the Global Menstrual Collective (www.globalmenstrualcollective.org). The Global Menstrual Collective was established in 2019 to bring together multi-sectoral stakeholders and coalitions working on menstrual health with the purpose of supporting coordination and bolstering collective, evidence-based advocacy to drive investment. The Terminology Action Group (see author contributions), which includes members reflecting research, practice, advocacy and funding perspectives, developed an initial draft definition drawing on past applications of terms such as “menstrual health” and “menstrual hygiene” in research and practice documentation,^6,8,9^ the World Health Organization’s definition of health, and the recent Lancet-Guttmacher Commission’s definition of Sexual and Reproductive Health and Rights (SRHR).^10^ This draft definition was shared with key stakeholders identified through membership of the Global Menstrual Collective,^4^ regional coalitions, and organisations with a history of funding or programming for menstrual health. Stakeholders completed a standardised form which requested feedback on: (1) alignment with the WHO definition of health, (2) the requirements for achieving menstrual health outlined as part of the definition, and (3) the specific wording of the definition itself. An overwhelming majority endorsed the approach and provided feedback to refine the definition statement. Fifty-one experts provided detailed responses, representing academic institutions (18%), non-governmental organisations (27%), UN agencies (24%), funding organisations (8%), government (4%), regional coalitions focused on menstrual health (14%), and professional associations for gynaecological health (6%). Stakeholders were from organisations based in Europe (29%), the Americas (25%), Africa (25%) and South and South-East Asia (20%).

## Defining menstrual health

Box 1 presents the definition of menstrual health developed by the Terminology Action Group.

**Box 1. Definition of menstrual health**

Menstrual health is a state of complete physical, mental, and social well-being and not merely the absence of disease or infirmity, in relation to the menstrual cycle.

Achieving menstrual health implies that women, girls, and all other people who experience a menstrual cycle, throughout their life-course, are able to:

- access accurate, timely, age-appropriate information about the menstrual cycle, menstruation, and changes experienced throughout the life-course, as well as related self-care and hygiene practices.
- care for their bodies during menstruation such that their preferences, hygiene, comfort, privacy, and safety are supported. This includes accessing and using effective and affordable menstrual materials and having supportive facilities and services, including water, sanitation and hygiene services, for washing the body and hands, changing menstrual materials, and cleaning and/or disposing of used materials.
- access timely diagnosis, treatment and care for menstrual cycle-related discomforts and disorders, including access to appropriate health services and resources, pain relief, and strategies for self-care.
- experience a positive and respectful environment in relation to the menstrual cycle, free from stigma and psychological distress, including the resources and support they need to confidently care for their bodies and make informed decisions about self-care throughout their menstrual cycle.
- decide whether and how to participate in all spheres of life, including civil, cultural, economic, social, and political, during all phases of the menstrual cycle, free from menstrual-related exclusion, restriction, discrimination, coercion, and/or violence.

This definition of menstrual health aligns with the WHO definition of health and attends to mental and social, as well as physical well-being. We intentionally link menstrual health to the menstrual cycle. This acknowledges that menstrual-related discomforts and disorders, consequences for mental well-being, and social exclusion are not restricted to the menstrual period. Whilst the majority of those who experience a menstrual cycle are women and girls, this approach also communicates the relevance of menstrual health for all those who experience a menstrual cycle, regardless of their gender identity. Further, it recognises that many who experience a menstrual cycle may not experience regular bleeding and the absence of menstruation can be a source of anxiety and distress.^11^

Referring to “women, girls, and all other people who experience a menstrual cycle” draws attention to the fact that people experience menstruation differently, shaped by their lived experiences, needs and circumstances. Disability, age, gender identity, place of residence, homelessness, housing instability, conditions of detention, migration, disaster, insecurity and displacement, religion, ethnicity, caste, culture and many other factors influence menstrual experiences and must be considered to adequately meet menstrual health needs.^12–15^ This does not mean that those who do not experience a menstrual cycle are not affected by social, cultural and economic aspects of menstruation or that they should not play an essential role in achieving this state for others. On the contrary, achieving a complete state of menstrual health requires education about the menstrual cycle for everyone, including men and boys,^16–19^ health care providers,^20,21^ and the dismantling of harmful stigma and norms amongst society at large.^22–24^

Our core definition of menstrual health is followed by requirements for achieving this state. Requirements listed in bullet points include: information about the menstrual cycle and self-care; materials, facilities and services to care for the body during menstruation; diagnosis, care, and treatment for menstrual discomforts and disorders; a positive and respectful environment which minimises psychological distress; and freedom to participate in all spheres of life. Below we elaborate on each of these needs and their importance for menstrual health to support interpretation of the definition.

### Information

To ensure menstrual health, women, girls, and others who experience a menstrual cycle must have access to accurate biological and practical information. Biological information about the menstrual cycle and its relationship to reproduction and fertility enables understanding of the body for menstrual health and SRHR.^11,25^ Practical knowledge, such as information about hygiene, nutrition, and self-care, equips those who menstruate to make informed decisions,^26^ supports alleviating discomforts, and bodily autonomy.^24,27^ Further, accurate information can dismantle misconceptions and taboos which compromise menstrual health.^24,28^ The provision of this information must be timely to support mental well-being and equip people who experience a menstrual cycle to determine which changes are normal and which might require medical attention. For example, information about menstruation must be provided prior to menarche. Similarly, knowledge about changes to the menstrual cycle due to contraceptive methods,^11^ pregnancy and the post-partum period, perimenopause, menopause, and in response to disease or other health stressors such as substantial weight loss is required before the onset of these changes. Finally, for information to be accessible and understood it needs to be age-appropriate and in formats for people with different impairments.^7,29,30^

### Materials, facilities, and services

To support menstrual health, individuals must be able to select care practices that are preferable and comfortable for them, and be able to afford the resources required for self-care.^7^ These practices should support hygiene and minimise the risk of infection and harm. Women, girls, and others who menstruate must be able to care for their body with the level of privacy they desire such that they feel free from unwanted observation or disturbance,^31^ and in safety such that they are protected from risk of physical, emotional or social harm. Safety must be considered in the location of infrastructure and services, the quality of menstrual materials, infrastructure, and disposal practices. The menstrual health of the individual requires that disposal practices protect from emotional and social harm, while disposal practices are also contributors to environmental health.^32^ Research has identified a broad range of practices undertaken by individuals to care for their body during menses, and the infrastructure and services required to support these.^8,9,33,34^ Self-care needs are not limited to accessing materials to collect menses, but include transporting and storing materials, and require facilities and services for changing materials, washing hands and the body, disposing of used materials and cleaning reusable materials which may include washing, drying and other sterilising practices such as ironing or boiling.^7,33^ These care needs are relevant throughout the day and night, both at and away from the home. Materials, facilities, and services need to be accessible to people with disabilities. Difficulties managing menstruation are a source of distress,^7^ irritation and discomfort, have been identified as barriers to education and employment, have been linked to potential reproductive tract infections,^35–37^ and can compromise social well-being.^7,30,38^

### Diagnosis, care and treatment for discomforts and disorders

A range of disorders of the menstrual cycle have significant implications for physical, mental, and social well-being.^39^ In addition, other difficulties associated with the menstrual cycle impact quality of life including pain, physical discomfort, impacts on mental health,^40,41^ and abnormal uterine bleeding.^42^ These may occur in the absence of disorders of the menstrual cycle. Timely diagnosis and support for disorders and discomforts requires those experiencing a menstrual cycle to be able to identify menstrual symptoms that are abnormal for their body, to feel comfortable seeking advice and support, and to have access to health services provided by competent health workers who operate in a system that is responsive to menstrual health needs.^39^ In grounding menstrual health within the menstrual cycle, we recognise that the treatment of health conditions that may cause abnormal uterine bleeding, such as uterine fibroids or cancer, falls beyond the remit of menstrual health. However, by addressing menstrual health needs there is significant opportunity to improve the health of those experiencing these conditions.^43^ Treatment and care for discomforts and disorders may range from clinical care to advice for self-care or access to resources such as medication, counselling, exercise, or heat therapy.^44–46^

### A positive and respectful environment

Harmful norms and stigma surrounding the menstrual cycle undermine physical, mental, and social well-being.^47,48^ A positive and respectful environment is needed across all levels, including the interpersonal, community and societal, for individuals to attain and maintain menstrual health. This means menstrual health must be considered in decision-making to ensure policies and programmes support a safe and positive environment. Resources and support may be required from a variety of sources, such as family members, care-givers, the community, educational institutions and the government, to equip individuals to care for their body with confidence throughout their menstrual cycle.^7^ Making informed decisions about menstrual care requires that people experiencing a menstrual cycle have access to sufficient information about the available options (addressed in item 1) and are empowered to make their own decisions based on their values.^49^ Such decisions range from the selection of safe, acceptable menstrual materials, to accessing health care for discomforts and disorders.

### Freedom to participate in all spheres of life

Social well-being, as part of menstrual health, requires that individuals are free to choose to participate in civil, cultural, economic, social, and political life without restrictions or exclusions related to their menstrual cycle. We emphasise decision-making and choice by menstruators who may decide whether to engage in activities depending on their preferences, values, and beliefs. Individuals may choose to abstain from participation, and we recognise that menstrual-related restrictions may be preferred. In other cases, social expectations and coercion linked to menstruation exclude preferred participation, with negative repercussions for physical, mental, and social well-being.^7^ Persistent lack of power in decision-making combined with harassment, violence or exclusion may have further negative impacts on health and safety. Freedom to participate in all spheres of life across the menstrual cycle has implications for many other human rights beyond the right to health, including education, work, and culture.

## Discussion

The definition of menstrual health presented here reflects current evidence and practice. The past decade has seen exponential growth in attention to menstrual health, and as the field continues to expand, we expect that new iterations of the definition may be needed. Given the multifaceted nature of menstrual health, expanded components of the definition and other terms may be useful for dialogue and advocacy within each sector and for informing action and research.

This definition of menstrual health builds on the foundation laid by advocates for menstrual health and hygiene. In 2012, “menstrual hygiene management” was defined by the Joint Monitoring Program (JMP) for Water Supply, Sanitation and Hygiene as

*“Women and adolescent girls using a clean menstrual management material to absorb or collect blood that can be changed in privacy as often as necessary for the duration of the menstruation period, using soap and water for washing the body as required, and having access to facilities to dispose of used menstrual management materials. They understand the basic facts linked to the menstrual cycle and how to manage it with dignity and without discomfort or fear.”*^50^

This definition was developed to advocate for the inclusion of menstrual needs in the Sustainable Development Goals. Menstrual hygiene was also included in the recent Lancet-Guttmacher definition of reproductive health.^10,51^ As the field has grown, however, there is a recognised need for new terminology that (1) avoids unintentionally reinforcing menstruation as dirty or impure, which has been voiced as a critique of “hygiene” terminology,^5,52^ (2) goes beyond the care of menstrual bleeding to include the many social and psychological components of menstrual experience, as well as needs related to health and social inclusion, and (3) is inclusive of gender-diverse populations.^6^ The term “menstrual health” has been used by a range of stakeholders in the absence of a self-contained or established definition. The most frequently used explanation of the meaning of “menstrual health” has been as an extension of menstrual hygiene to “other systematic factors linking menstruation with health and wellbeing”.^6,9^

The definition we provide here builds on and incorporates the essential contributions made by past work and seeks to evolve terminology in research and practice. It offers a concise statement aligned with the WHO’s definition of health, in addition to the requirements needed to attain menstrual health. We have chosen this approach to position the field for greater recognition and engagement with other actors in the health and gender communities.

## Conclusions and use of the definition

As the Global Menstrual Collective Terminology Action Group, we offer a new definition of menstrual health that benefits from the foundations of past work, and consultation with expert stakeholders. We encourage the adoption of this definition to unify, guide and inform advocacy, policy, practice and research. The definition can be used to situate menstrual health across sectoral priorities and funding portfolios for other health priorities such as SRHR, WASH, and adolescent and women’s health. This unified definition offers a point of consolidation and foundation for partnerships to address the broad scope of this challenge. The definition can also be used to drive innovations for improving menstrual health and develop priorities and indicators against which to monitor progress.

To achieve health and equality for all we can no longer stigmatise menstruation with innuendo or neglect the needs of those who menstruate by requiring they fit neatly into other existing health priorities and budget lines. It is time for menstrual health to receive attention and investment commensurate with its importance in the lives of the billions of people who menstruate.

## Data Availability

All data are contained within the manuscript and supporting files.
